# Supplementary figures and images for: Metabolic coupling of ROS generation and antioxidant synthesis by the GABA shunt pathway in myeloid-like blood progenitor cells of Drosophila
Source: PLoS Genet. 2025 Sep 29;21(9):e1011602. doi: 10.1371/journal.pgen.1011602 (PMC12500130; doi:10.1371/journal.pgen.1011602)

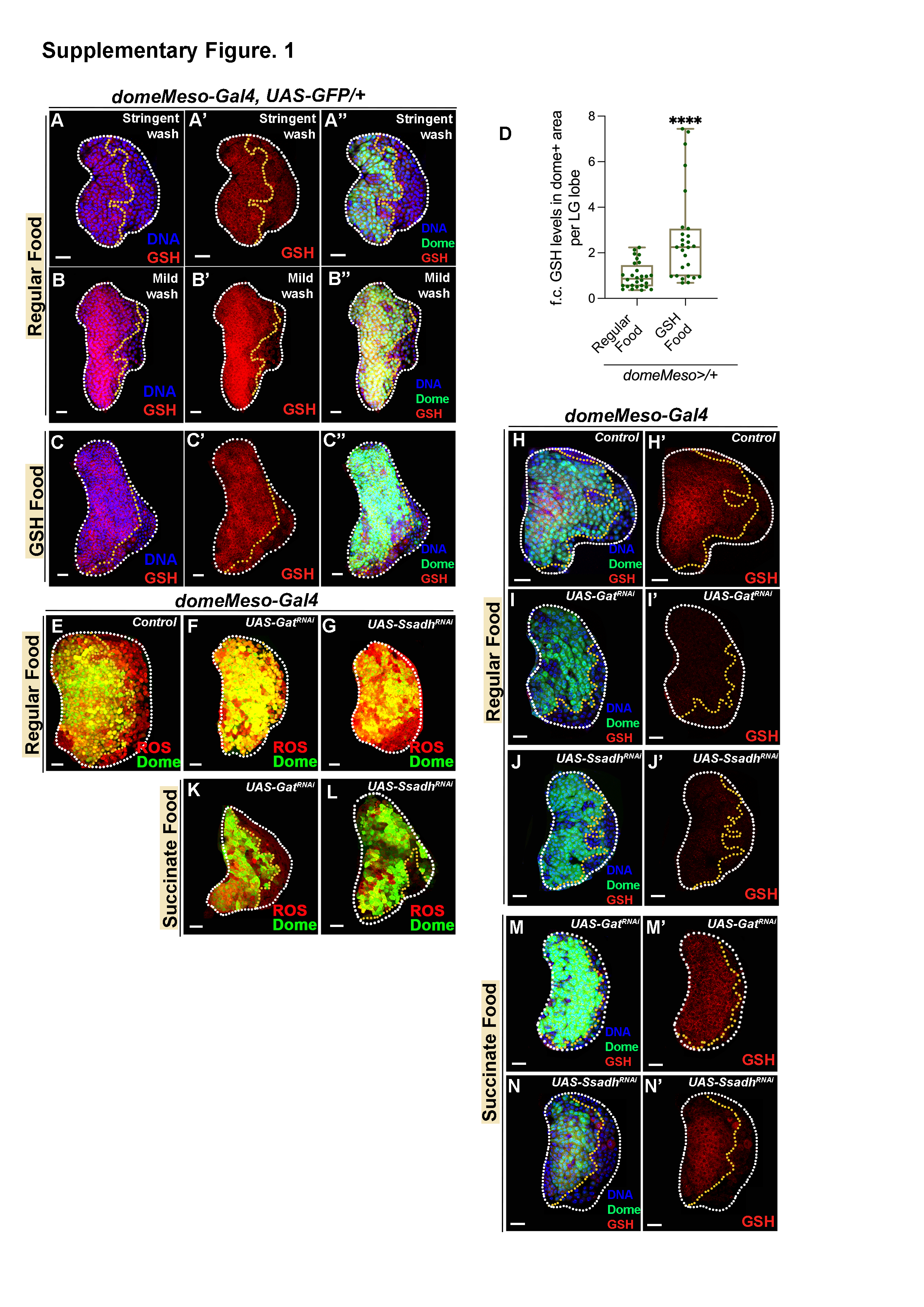

Supplement: S1 Fig — RF is regular food, and GSH food and succinate food is GSH and succinate supplemented food respectively. Data is presented as median plots (****p < 0.0001). Mann-Whitney test is applied for D. In D, ‘n’ is total number of lymph gland lobes analysed and is represented by a green dot. Scale bar: 20µm. DNA is stained with DAPI in blue, dome marks the progenitor cells in green. Comparisons for significance are done with their respective control. White border demarcates the lymph gland lobe and yellow border marks the dome positive area towards the left side. (A-C”) Representative images showing GSH levels in the lymph gland progenitor cells shown by merge of dome+ (green), DNA (blue) and GSH (red) from domeMeso-Gal4,UAS-GFP/+ genetic backgrounds upon different washing conditions. (A-A”) GSH (red) levels in the progenitor cells upon stringent washing with 0.3% triton x-100 show a reduced expression with distinction of GSH levels in the medullary and cortical zone and (B-B”) GSH (red) levels in the progenitor cells upon mild washing with 0.1% tween-20 show an elevated expression, (C-C”) supplementing control animals with GSH (GSH, domeMeso-Gal4,UAS-GFP/+) and utilization of stringent washing protocol leads to a significant increase in GSH levels in the progenitor cells. For comparison, refer to control (A-A”, Regular Food). For quantifications of GSH levels in regular food and GSH supplementation, refer to D. (D) Quantification of blood-progenitor GSH levels in domeMeso>GFP/+ (control, Regular Food, n=28), and domeMeso>GFP/+ (GSH Food, n=27, p<0.0001). (E-G) Representative images showing ROS levels in the lymph gland progenitor cells shown by merge of dome+ (green) and ROS (red) from different genetic backgrounds. Control (domeMeso-Gal4,UAS-GFP/+) showing elevated (E) ROS (stained with DHE, red) levels in the lymph gland progenitor cells, expressing (F) GatRNAi (domeMeso-Gal4,UAS-GFP;UAS-GatRNAi) and (G) SsadhRNAi (domeMeso-Gal4,UAS-GFP;UAS-SsadhRNAi) leads to an increase [file pgen.1011602.s001.tif]

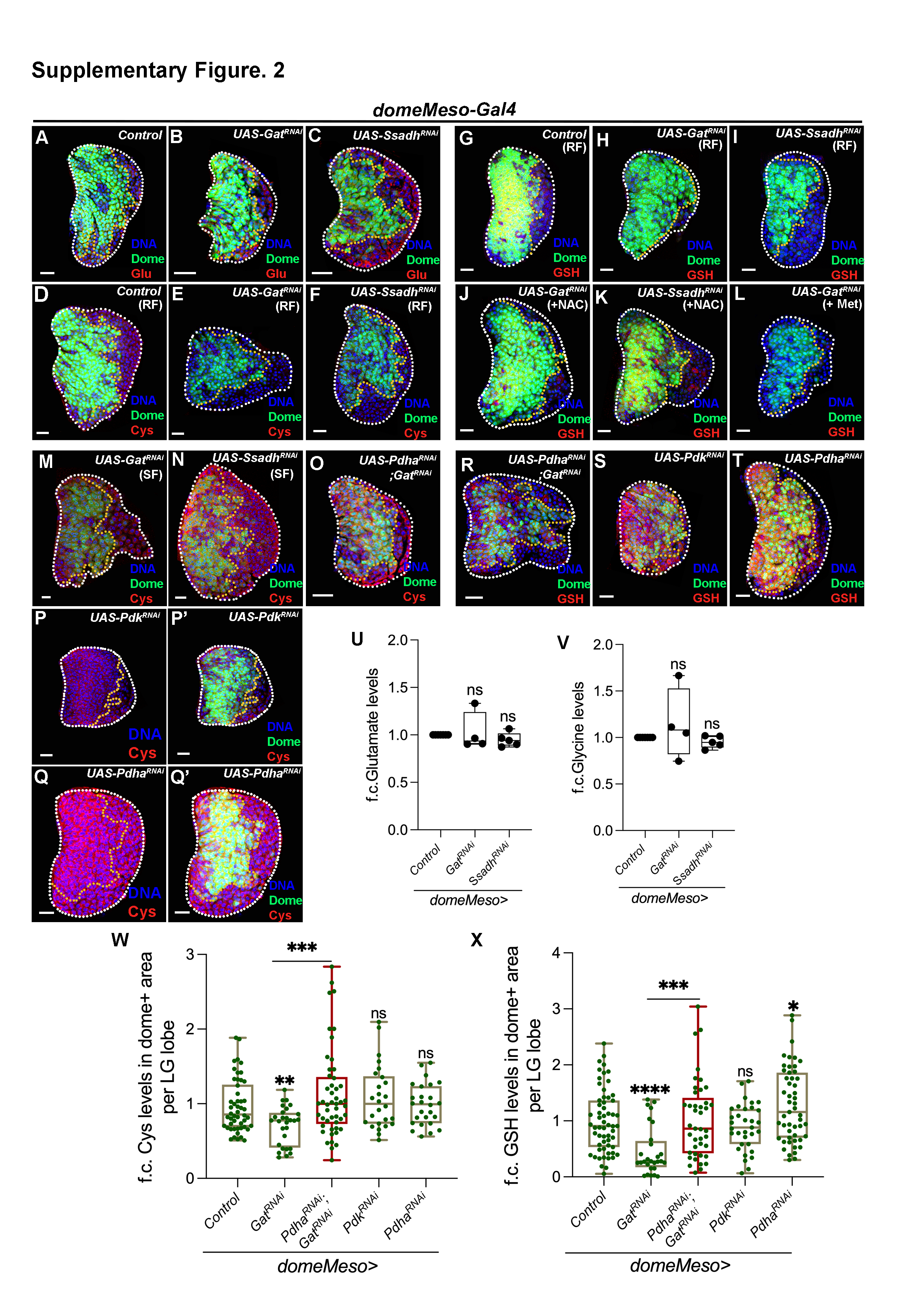

Supplement: S2 Fig — RF is regular, SF is succinate, NAC is N-acetylcysteine and Met is methionine supplemented food. Data is presented as median plots (*p < 0.05; **p < 0.01; ***p < 0.001; ****p < 0.0001 and ns is non-significant). T-test is applied for U, V and Mann-Whitney test is applied for W, X. In U, V ‘n’ is sample size and ‘N’ is number of experimental repeats and shown by black dot. In W, X ‘n’ is total number of lymph gland lobes analysed and is represented by a green dot. Scale bar: 20µm. DNA is stained with DAPI in blue, dome marks the progenitor cells in green. Comparisons for significance are done with control and with respective genetic conditions for rescue combinations (red bars), which are indicated by horizontal lines drawn above the box plots. White border demarcates the lymph gland lobe and yellow border marks the dome positive area towards the left side. (A-C) Representative images showing glutamate levels in lymph gland progenitor cells (area marked within the yellow dotted line) with merge of dome+ (green), DNA (blue) and glutamate (Glu, red) across different genetic backgrounds. (A) Control (domeMeso-Gal4,UAS-GFP/+) lymph gland showing relatively uniform glutamate levels across all cells of the tissue, including the progenitor-cells (area within the yellow dotted line). Expressing (B) GatRNAi (domeMeso-Gal4,UAS-GFP;UAS-GatRNAi) and (C) SsadhRNAi (domeMeso-Gal4,UAS-GFP;UAS-SsadhRNAi) in the progenitor cells does not affect their glutamate levels in comparison to control (A). (D-F) Representative images showing cysteine (Cys, red) levels in lymph gland progenitor cells (area marked within the yellow dotted line) with merge of dome+ (green), DNA (blue) and cysteine (red) from different genetic backgrounds. (D) Control (RF, domeMeso-Gal4,UAS-GFP/+) lymph gland showing relatively uniform cysteine levels in all cells of the lymph gland including progenitor-cells (area demarcated within the yellow border). Expressing (E) GatRNAi (RF, domeMeso-Gal4,UAS-GFP;UAS-GatRNAi) [file pgen.1011602.s002.tif]

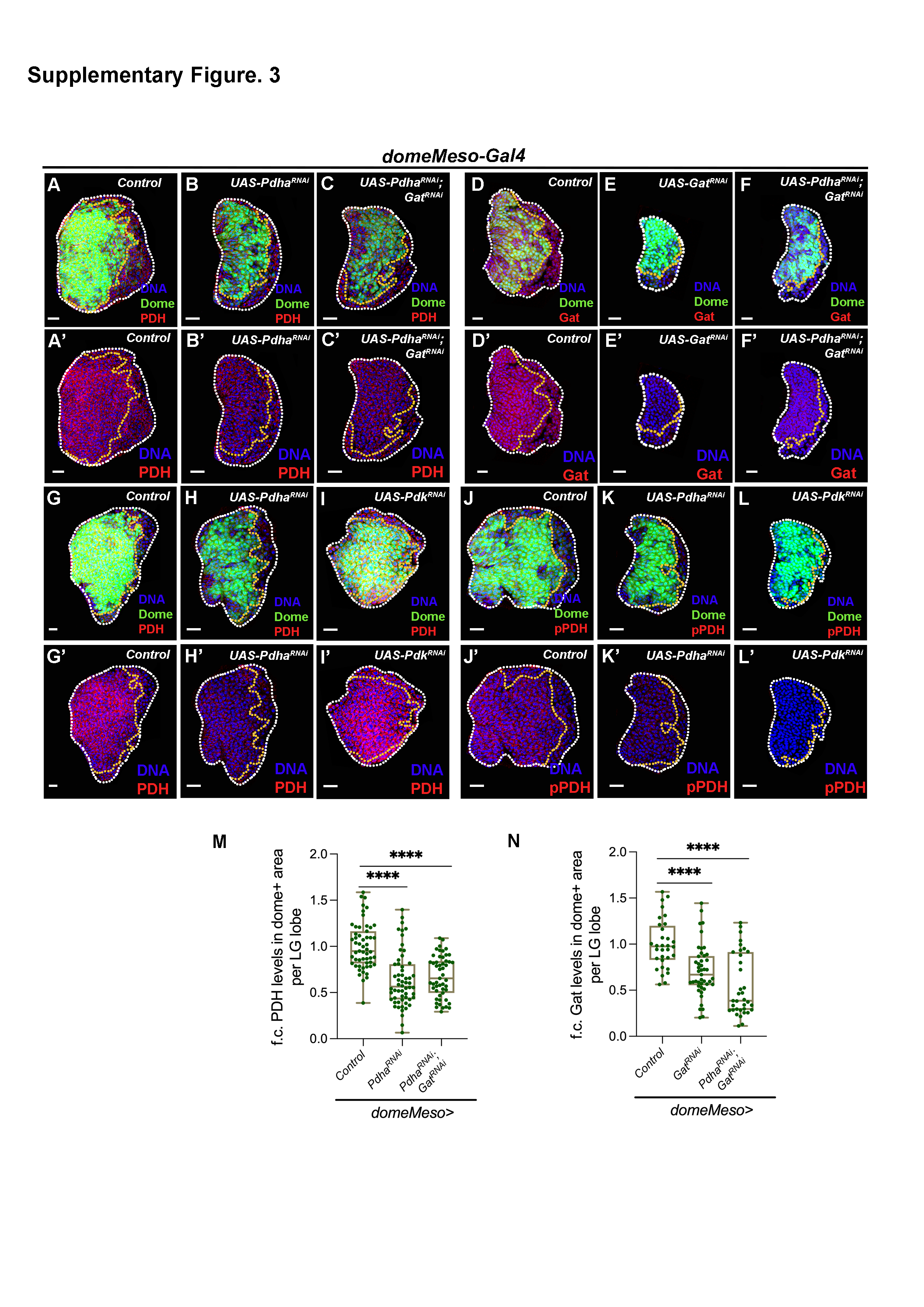

Supplement: S3 Fig — Data is presented as median plots (****p < 0.0001). Mann-Whitney test is applied for M, N and ‘n’ is total number of lymph gland lobes analysed and is represented by a green dot. Scale bar: 20µm. DNA is stained with DAPI in blue, dome marks the progenitor cells in green. Comparisons for significance are done with their respective control. White border demarcates the lymph gland lobe and yellow border marks the dome positive area towards the left side. (A-C’) Representative images showing PDH (red) levels in lymph gland progenitor cells (area marked within the yellow dotted line) with merge of dome+ (green), DNA (blue) and PDH (red) from different genetic backgrounds. (A, A’) Control (domeMeso-Gal4,UAS-GFP/+) lymph gland showing PDH levels, expressing (B, B’) PdhaRNAi (domeMeso-Gal4,UAS-GFP;UAS-PdhaRNAi) and (C, C’) PdhaRNAi;GatRNAi (domeMeso-Gal4,UAS-GFP; PdhaRNAi;GatRNAi) in the progenitor cells leads to reduction in PDH levels. For quantifications, refer to M. (D-F’) Representative images showing Gat (red) levels in lymph gland progenitor cells (area marked within the yellow dotted line) with merge of dome+ (green), DNA (blue) and Gat (red) from different genetic backgrounds. (D, D’) Control (domeMeso-Gal4,UAS-GFP/+) lymph gland showing Gat levels, expressing (E, E’) GatRNAi (domeMeso-Gal4,UAS-GFP;UAS-GatRNAi) and (F, F’) PdhaRNAi;GatRNAi (domeMeso-Gal4,UAS-GFP; PdhaRNAi;GatRNAi) in the progenitor cells leads to reduction in Gat levels. For quantifications, refer to N. (G-I’) Representative images showing PDH (red) levels in lymph gland progenitor cells (area marked within the yellow dotted line) with merge of dome+ (green), DNA (blue) and PDH (red) from different genetic backgrounds. (G, G’) Control (domeMeso-Gal4,UAS-GFP/+) lymph gland showing PDH levels, expressing (H, H’) PdhaRNAi (domeMeso-Gal4,UAS-GFP;UAS-PdhaRNAi) leads to reduction in PDH levels and expressing (I, I’) PdkRNAi (domeMeso-Gal4,UAS-GFP; PdkRNAi) in the progenitor cells does not show any change [file pgen.1011602.s003.tif]

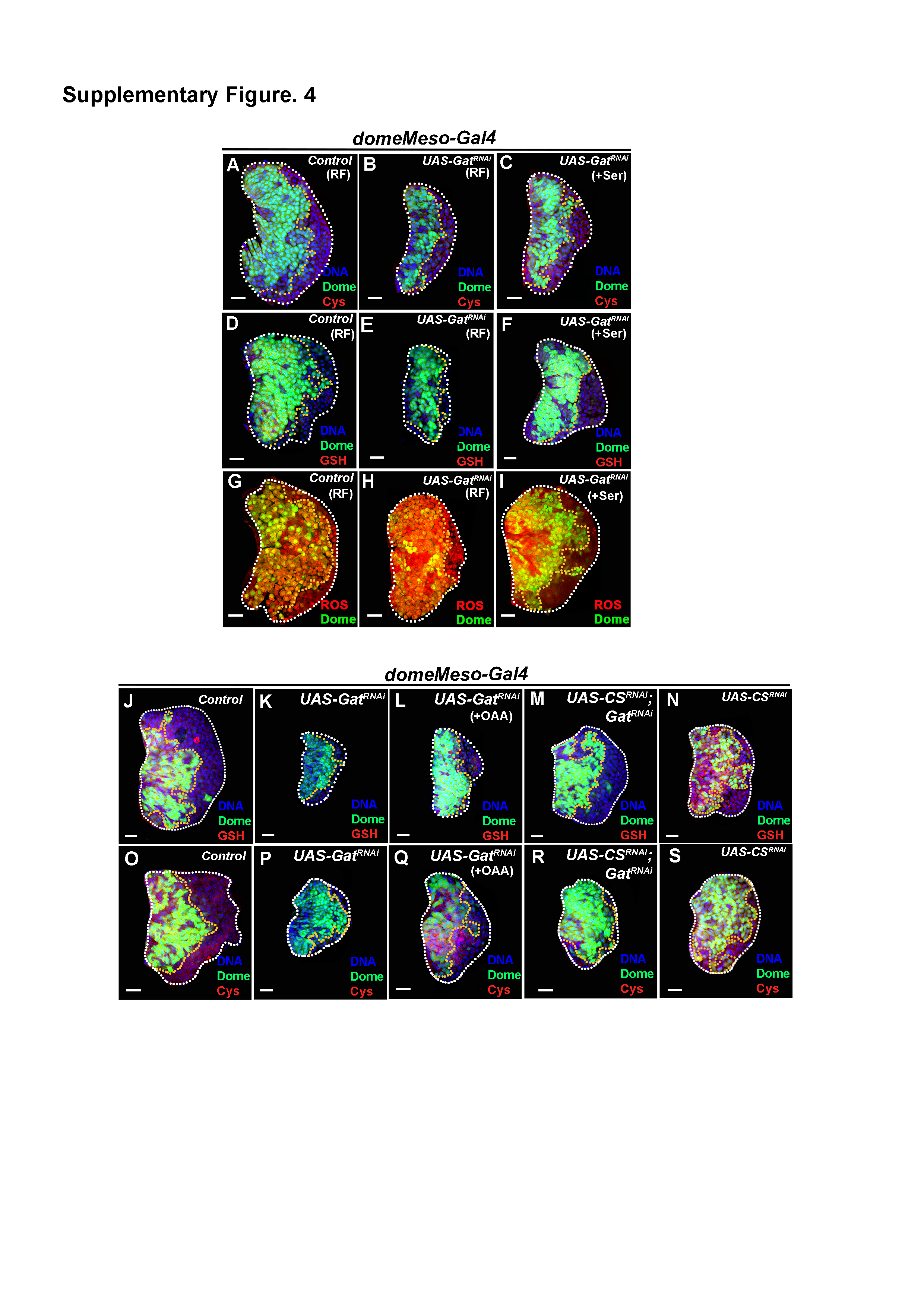

Supplement: S4 Fig — RF is regular food, Ser is serine and OAA is oxaloacetate supplemented food. Scale bar: 20µm. DNA is stained with DAPI in blue, dome marks the progenitor cells in green. White border demarcates the lymph gland lobe and yellow border marks the dome positive area towards the left side. (A-C) Representative images showing cysteine (Cys, red) levels in lymph gland progenitor cells (area marked within the yellow dotted line) with merge of dome+ (green), DNA (blue) and cysteine (red) from different genetic backgrounds. (A) Control (RF, domeMeso-Gal4,UAS-GFP/+) lymph gland showing relatively uniform cysteine levels in all cells of the lymph gland including progenitor-cells (area demarcated within the yellow border). While, expressing (B) GatRNAi (RF, domeMeso-Gal4,UAS-GFP;UAS-GatRNAi) in the progenitor cells leads to reduction in cysteine levels as compared to control (A), supplementing this genetic condition with (C) serine (Ser, domeMeso-Gal4,UAS-GFP;UAS-GatRNAi) recovers cysteine levels almost comparable to control (A). For comparison, also refer to (B) GatRNAi raised on regular food (RF). (D-F) Representative images showing GSH (red) levels in lymph gland progenitor cells (area marked within the yellow dotted line) with merge of dome+ (green), DNA (blue) and GSH (red) from different genetic backgrounds. (D) Control (RF, domeMeso-Gal4,UAS-GFP/+) lymph gland showing GSH levels in lymph gland progenitor-cells (area demarcated within the yellow border). While, expressing (E) GatRNAi (RF, domeMeso-Gal4,UAS-GFP;UAS-GatRNAi) in the progenitor cells leads to reduction in GSH levels as compared to control (D), supplementing this genetic condition with (F) serine (Ser, domeMeso-Gal4,UAS-GFP;UAS-GatRNAi) recovers GSH levels almost comparable to control (D). For comparison, also refer to (E) GatRNAi raised on regular food (RF). (G-I) Representative images showing ROS levels (area marked within the yellow dotted line) with merge of dome+ (green) and ROS (red) from different genetic [file pgen.1011602.s004.tif]

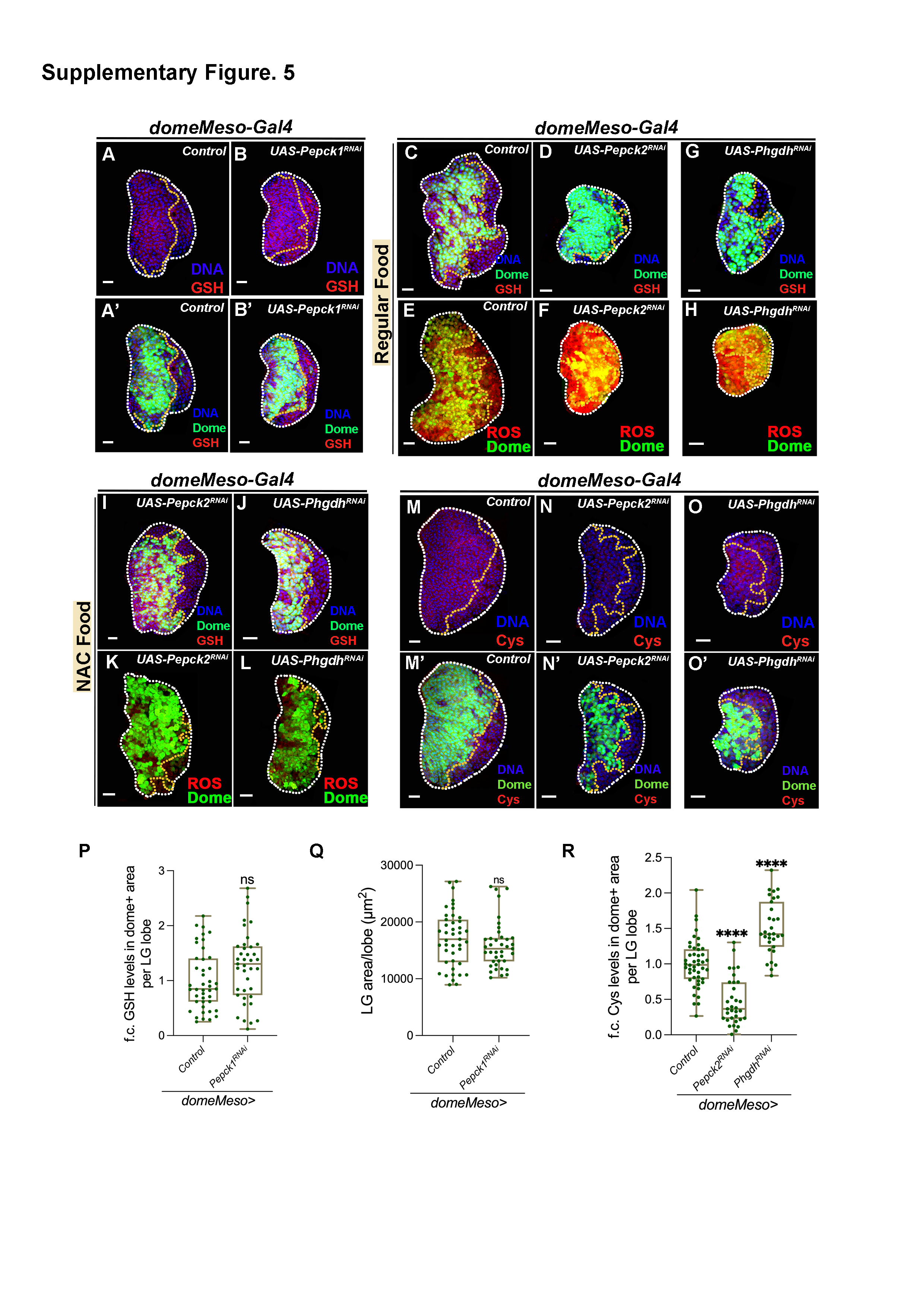

Supplement: S5 Fig — RF is regular food and NAC is N-acetylcysteine supplemented food. Data is presented as median plots (****p < 0.0001 and ns is non-significant). Mann-Whitney test is applied for P-R. In P-R, ‘n’ is total number of lymph gland lobes analysed and is represented by a green dot. Scale bar: 20µm. DNA is stained with DAPI in blue. Comparisons for significance are done with their respective control. White border demarcates the lymph gland lobe and yellow border marks the dome positive area towards the left side. (A-B’) Representative images showing glutathione (GSH, red) levels in lymph gland progenitor cells (area marked within the yellow dotted line) and with merge of dome+ (green), DNA (blue) and GSH (red) from different genetic backgrounds. In comparison to (A, A’) control (domeMeso-Gal4,UAS-GFP/+) lymph gland, expressing (B, B’) Pepck1RNAi (domeMeso-Gal4,UAS-GFP;UAS-Pepck1RNAi) in the progenitor cells does not show any change in blood-progenitor GSH levels. For quantifications, refer to P. (C-L) Representative images showing glutathione (GSH, red) and ROS (red) levels in lymph gland progenitor cells with merge of dome+ (green), DNA (blue) and GSH/ROS (red) from different genetic backgrounds. (C) control (domeMeso-Gal4,UAS-GFP/+) lymph gland showing GSH levels, expressing (D) Pepck2RNAi (domeMeso-Gal4,UAS-GFP;UAS-Pepck2RNAi) leads to reduction in blood-progenitor GSH levels. (E) control (domeMeso-Gal4,UAS-GFP/+) lymph gland showing ROS levels, expressing (F) Pepck2RNAi (domeMeso-Gal4,UAS-GFP;UAS-Pepck2RNAi) leads to elevation of blood-progenitor ROS levels and similarly expressing (G, H) PhgdhRNAi (domeMeso-Gal4,UAS-GFP;UAS-PhgdhRNAi) leads to reduction in blood-progenitor (G) GSH levels and increase in (H) ROS levels. Compare to control (C) GSH and (E) ROS. NAC supplementation to (I, K) Pepck2RNAi (NAC, domeMeso-Gal4,UAS-GFP;UAS-Pepck2RNAi) and (J, L) PhgdhRNAi (NAC, domeMeso-Gal4,UAS-GFP;UAS-PhgdhRNAi) leads to recovery of blood progenitor (I, J) GSH and (K, L) ROS le [file pgen.1011602.s005.tif]

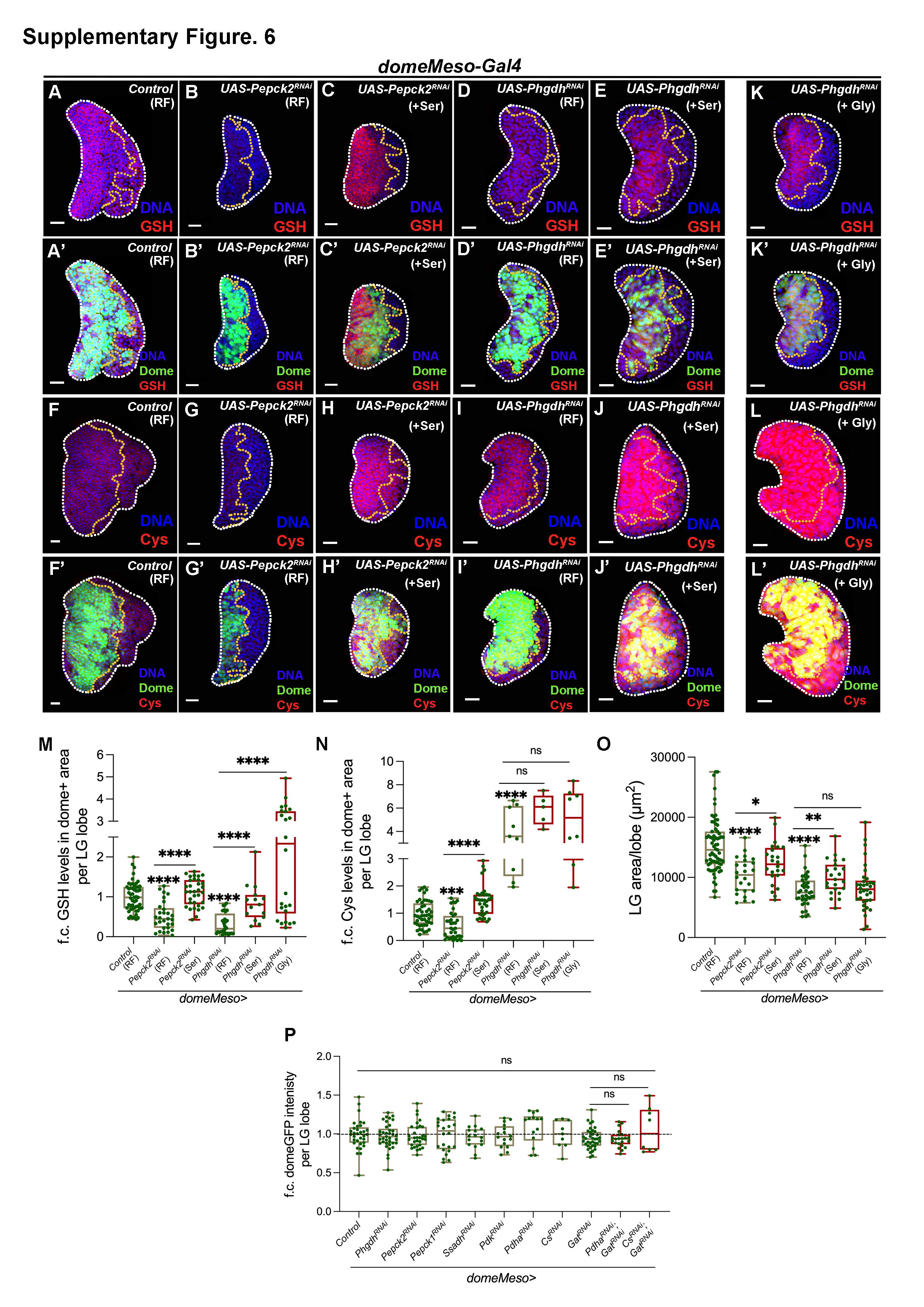

Supplement: S6 Fig — RF is regular food, Ser is serine and Gly is glycine supplemented food. Data is presented as median plots (*p < 0.05; **p < 0.01; ***p < 0.001; ****p < 0.0001 and ns is non-significant). Mann-Whitney test is applied for M-P. In M-P, ‘n’ is total number of lymph gland lobes analysed and is represented by a green dot. Scale bar: 20µm. DNA is stained with DAPI in blue, dome marks the progenitor cells in green. Comparisons for significance are done with control and with respective genetic conditions for rescue combinations (red bars), which are indicated by horizontal lines drawn above the box plots. White border demarcates the lymph gland lobe and yellow border marks the dome positive area towards the left side. (A-F’) Representative images showing glutathione (GSH, red) levels in lymph gland progenitor cells (area marked within the yellow dotted line) and with merge of dome+ (green), DNA (blue) and GSH (red) from different genetic backgrounds. In comparison to (A, A’) control (RF, domeMeso-Gal4,UAS-GFP/+) lymph gland, expressing (B, B’) Pepck2RNAi (RF, domeMeso-Gal4,UAS-GFP;UAS-Pepck2RNAi) leads to reduction in blood-progenitor GSH levels as compared to control (A, A’) and (C, C’) serine supplementation in Pepck2RNAi (Ser, domeMeso-Gal4,UAS-GFP;UAS-Pepck2RNAi) leads to recovery of blood progenitor GSH levels as compared to (B, B’) Pepck2RNAi on RF, expressing (D, D’) PhgdhRNAi (RF, domeMeso-Gal4,UAS-GFP;UAS-PhgdhRNAi) leads to reduction in blood-progenitor GSH levels as compared to control (A, A’) and (E, E’) serine supplementation in PhgdhRNAi (Ser, domeMeso-Gal4,UAS-GFP;UAS-PhgdhRNAi) recovers blood-progenitor GSH levels as compared to (D, D’) PhgdhRNAi on RF. For quantifications, refer to M. (F-J’) Representative images showing Cys (red) levels in lymph gland progenitor cells (area marked within the yellow dotted line) and with merge of dome+ (green), DNA (blue) and Cys (red) from different genetic backgrounds. In comparison to (F, F’) control (RF, domeMeso-Gal4,UA [file pgen.1011602.s006.tif]
