# Supplementary material for: Metabolic coupling of ROS generation and antioxidant synthesis by the GABA shunt pathway in myeloid-like blood progenitor cells of Drosophila
Source: PLoS Genet. 2025 Sep 29;21(9):e1011602. doi: 10.1371/journal.pgen.1011602 (PMC12500130; doi:10.1371/journal.pgen.1011602)
Supplement: S1 Table — (DOCX) [file pgen.1011602.s007.docx]

| **Analyte** | **Q1/Q3** | **Retention Time (R_T_)** |
| --- | --- | --- |
| αKG [m+0] | 462.1 -> 91.1 | 6.224 |
| αKG [m+1] | 463.1 -> 91.1 | 6.224 |
| αKG [m+2] | 464.1 -> 91.1 | 6.224 |
| αKG [m+3] | 465.1 -> 91.1 | 6.224 |
| αKG [m+4] | 466.1 -> 91.1 | 6.224 |
| αKG [m+5] | 467.1 -> 91.1 | 6.224 |
| Citrate [m+0] | 508.1 -> 91.1 | 5.329 |
| Citrate [m+1] | 509.1 -> 91.1 | 5.329 |
| Citrate [m+2] | 510.1 -> 91.1 | 5.329 |
| Citrate [m+3] | 511.1 -> 91.1 | 5.329 |
| Citrate [m+4] | 512.1 -> 91.1 | 5.329 |
| Citrate [m+5] | 513.1 -> 91.1 | 5.329 |
| Citrate [m+6] | 514.1 -> 91.1 | 5.329 |
| Malate [m+0] | 345.1 -> 91.1 | 4.207 |
| Malate [m+1] | 346.1 -> 91.1 | 4.207 |
| Malate [m+2] | 347.1 -> 91.1 | 4.207 |
| Malate [m+3] | 348.1 -> 91.1 | 4.207 |
| Malate [m+4] | 349.1 -> 91.1 | 4.207 |
| OAA [m+0] | 448.1 -> 91.1 | 7.357 |
| OAA [m+1] | 449.1 -> 91.1 | 7.357 |
| OAA [m+2] | 450.1 -> 91.1 | 7.357 |
| OAA [m+3] | 451.1 -> 91.1 | 7.357 |
| OAA [m+4] | 452.1 -> 91.1 | 7.357 |
| Serine | 106.2 -> 60.1 | 11.581 |
| Glutamate | 148.2 -> 130.2 | 20.557 |
| Glycine | 76.1 -> 30.1 | 10.598 |

**S1 Table.** Q1/Q3 and R_T_ values for the metabolites detected.
