# Supplementary material for: Metabolic coupling of ROS generation and antioxidant synthesis by the GABA shunt pathway in myeloid-like blood progenitor cells of Drosophila
Source: PLoS Genet. 2025 Sep 29;21(9):e1011602. doi: 10.1371/journal.pgen.1011602 (PMC12500130; doi:10.1371/journal.pgen.1011602)
Supplement: Attachment — Submitted filename: Goyal et al_Point by point Response to Reviewers.docx [file pgen.1011602.s010.docx]

***Response to Reviewer comments***

**We thank all the reviewers for their supportive and constructive feedback and overall very useful comments on our manuscript. We have now addressed all the concerns with respect to RNA*i* efficiency, statistical analysis and providing more data to strengthen the model in context of involvement of gluconeogenesis as suggested by the reviewers and strongly feel that with these changes the manuscript has improved in its overall quality and also clearer in terms of the model that is now being proposed.**

**We address all comments in the point-by-point response here with.**

Reviewer's Responses to Questions

Reviewer #1: The authors discovered that GSH is synthesized in blood progenitor cells in Drosophila lymph glands, and they investigated how GSH was generated in progenitor cells. Interestingly, antioxidant GSH was produced in cells producing ROS, which has been previously shown to be essential to maintaining the properties of progenitor cells. However, the authors did not address whether GSH production is crucial to maintaining ROS homeostasis or the properties of the blood progenitor cells. Thus, the paper describes possible pathways that generate GSH in the blood progenitor cells. There are problems with the interpretation of results as described below.

**We acknowledge the reviewers positive critique of our work and appreciate all comments made. In the revised version, we have made a strong attempt to address all concerns and the nature of experimentation or changes done are listed herewith.**

Major concerns:

1.Quantification of free amino acids: The authors quantified free amino acids based on the signal intensity of immunohistochemical staining. The method is not generally used, and I would like to know whether it is possible to fix free amino acids in tissues so they won't be washed away during the staining procedure. It would be helpful if the authors could provide any data or references to support the reliability of the methods.

**We appreciate the reviewer’s insightful comment with respect to use of immunohistochemical ways to detect small metabolites. Indeed it is critical to consider the potential loss of small molecules like amino acids during staining procedures, however, the use of antibodies to detect amino acids or their derivatives is well-established in various tissues, including the brain where immunohistochemical detection of neurotransmitters such as GABA, glutamate, and dopamine is routinely used to label GABAergic (Das et al. 2011; Zhao et al. 2022), glutamatergic (Kolodziejczyk et al. 2008), and dopaminergic (Brazdis et al. 2020; Chabrat et al. 2019) neurons.**

**Relevant to our system, prior studies have successfully employed immunostaining to detect extracellular and intracellular amino acids in blood tissue. Notably, GABA has been shown to be neuronally derived and detected in *Drosophila* blood progenitors (Madhwal et al. 2020; Shim et al. 2013), while dopamine, synthesized within blood progenitors, was detected via immunostaining (Kapoor et al. 2022). These precedents support the feasibility and specificity of our approach.**

**Recognizing the sensitivity of such staining, the use of optimized protocol to avoid loss of free amino acids by employing required detergent conditions (using milder detergent, Tween-20 instead of Triton X-100) has been the approach. We have utilized the same protocol and this precaution has been critical for reliable detection of amino acids in the past and also utilized in the current study for GSH, cysteine, and glutamate in the lymph gland. Details of this optimized protocol are referenced and now clearly described in the Methods section.**

**Additionally, to directly address the concern by the reviewer, we have added a new supplementary figure panel (Fig. S1A-B”) that illustrates the impact of mild versus stringent washes on GSH staining intensity. This supports the reliability of our method and highlights the importance of tailored protocols when detecting small molecules via immunohistochemistry. The respective text is now added in results (line 197-202) and methods (line 596-598) section.**

2) RNA*i* experiments. The authors performed RNAi-mediated gene knockdown. The efficiency of knockdown depends on the constructs, and it is essential to know the extent of downregulation to interpret the results. The efficiency was also affected by the number of UAS transgenes expressed simultaneously. I understand that UAS-GFP was always included in all RNA*i* experiments to visualize the population of progenitor cells. Therefore, two or three UAS constructs were under the control of GAL4. Since the amount of GAL4 protein is limited, RNA*i* efficiency would be variable depending on the number of UAS constructs. Quantification of gene expression levels is necessary in knockdown experiments.

**RNA*i* Efficiency Concerns:** **For this important point, to directly evaluate RNA*i* efficiency and address the concern of potential *Gal4* dilution due to the inclusion of multiple *UAS* constructs, we performed immunohistochemical analyses using antibodies against Gat, PDH (total), and phosphorylated PDH (pPDH). These were assessed across the following genotypes: domeMeso>GFP/+ (control), domeMeso>GFP/Pdha^RNAi^, domeMeso>GFP/Gat^RNAi^ and domeMeso>GFP/Pdha^RNAi^; Gat^RNAi^.**

**Our analyses revealed a clear reduction in Gat and PDH protein levels in the respective single knockdown conditions. Importantly, in the double knockdown condition (Pdha^RNAi^;Gat^RNAi^), both proteins were downregulated to a comparable extent, indicating that the presence of multiple *UAS* constructs does not compromise knockdown efficiency. These data are now presented in Supplementary Figure 3A-C’ and 3D-F’, with quantifications shown in panels S3M and S3N.**

**Additionally, to assess whether *Gal4* activity was affected by the increased transgene load, we also quantified GFP fluorescence intensity across genotypes as a proxy for *Gal4*-driven expression. We observed no significant variation in GFP levels, indicating that *Gal4* availability remains sufficient even with multiple UAS constructs (Fig. S6P). Together, these data confirm robust and consistent RNA*i* efficiency across all experimental conditions.**

**These data are now provided in the results (line 255-262) and methods (line 568-573) section.**

3) Line 211-216: The authors downregulated Pdha in *Gat^RNAi^* condition, assessed for cysteine and GSH levels, and concluded that downregulated Pdha suppressed the GABA component RNA*i* phenotype. However, as I mentioned above, it is also possible that the downregulation of *Gat^RNAi^* was inefficient because of the co-expression of two RNA*i* constructs. Therefore, the role of Pdha in GSH and cysteine levels remains unclear.

**Interpretation of GSH and Cysteine Rescue in Double RNA*i*:** **As also stated above, we have validated RNA*i*** **efficiency in the double knockdown condition (domeMeso>GFP/Pdha^RNAi^;Gat^RNAi^, Supplementary Fig. S3A-F’, S3M-N) and the data indicate that the efficiency of knockdown is not compromised when both RNA*i* constructs are co-expressed. (line 255-262)**

**We have additional data providing new mechanistic insight: In homeostasis, we propose that it is the exit of oxaloacetate (OAA) from the TCA cycle and not PDH-mediated entry of pyruvate into the TCA cycle that regulates GSH and cysteine levels (Fig. 4F-Q and Fig. S4J-S). This exit point, controlled by citrate synthase (CS), is required to drive gluconeogenesis and regenerate pyruvate for serine biosynthesis—the precursor for cysteine and GSH. In Gat^RNAi^ progenitors, elevated PDH activity increases TCA cycle flux and inhibits OAA exit, thus impairing serine and GSH synthesis.**

**Importantly, in Gat^RNAi^ condition, inhibition of citrate synthase (via Cs^RNAi^) or supplementation with OAA restores GSH and cysteine levels. These findings support a model in which metabolic flux through the TCA cycle is redirected under homeostatic conditions to support antioxidant defense, and that disruption of this redirection in Gat^RNAi^ cells leads to GSH deficiency. These results are included in the revised manuscript (line 347-367).**

4) 221-234: The authors performed downregulation of Pdk or Pdha, and neither affected the GSH and cysteine levels. These data suggest two possibilities: 1)*^RNAi^* did not work efficiently. Verification of knockdown is necessary. 2) Both*^RNAi^* worked adequately, but neither is involved in regulating GSH and cysteine levels.

***Pdha^RNAi^* and *Pdk^RNAi^* Validation: We have validated the efficacy of Pdha^RNAi^ and Pdk*^RNAi^* both in our previous work (Goyal et al. 2022) and in the present study. Specifically, PDH protein levels (PDH TOTAL) were used as a readout for Pdha knockdown, and pPDH (phosphorylation at S293) was used as a functional readout for Pdk activity, as PDK phosphorylates and inactivates PDH (line 258-262).**

**As shown in Supplementary Fig. S3G-I’ and S3J-L’, domeMeso>Pdha^RNAi^ resulted in a marked decrease in total PDH, while domeMeso>Pdk^RNAi^ specifically reduced pPDH levels without affecting total PDH—consistent with effective knockdown of the intended targets.**

**Despite these effective knockdowns, neither manipulation altered GSH or cysteine levels. This, together with our new findings, reinforces the idea that the regulation of GSH biosynthesis is not determined by pyruvate entry into the TCA via PDH, but rather by the ability of the cycle to divert OAA toward gluconeogenesis and serine synthesis.**

**Further, in Gat^RNAi^ lymph glands, we observe increased PDH activity and enhanced TCA cycling, corroborated by isotope tracing experiments that show elevated labeling of downstream metabolites. This hyperactivity appears to prevent OAA exit, suppressing the production of serine, cysteine, and GSH. Notably, both Pdha^RNAi^ and Cs^RNAi^ restore GSH/cysteine levels in this context, indicating that metabolic control points downstream of PDH are key regulators of antioxidant synthesis.**

**Finally, our mass spectrometry data suggest that in wild-type progenitors, pyruvate preferentially enters the TCA via pyruvate carboxylase (PC), as opposed to PDH (Fig. 4A-E’). GABA metabolism plays a regulatory role by inhibiting PDH, thus facilitating OAA diversion toward gluconeogenesis and supporting serine-derived antioxidant production. Loss of GABA metabolism (e.g., in Gat^RNAi^) lifts this inhibition, increasing ROS production while diminishing GSH biosynthesis. These new findings are now included in the revised manuscript and strengthen our proposed model.**

**We hope these data will satisfy all concerns with respect to RNA*i* efficacies and regulation of GSH/cysteine synthesis. We thank the reviewer for having carefully gone over the data to bring these critical points up, which have helped us in revising the draft with additional data and experiments. To our satisfaction, the current draft is much stronger in its overall presentation of the data and model.**

Minor comments:

Lines 133-139. should be described in the Discussion

**This is done.**

Line 174-178. Progenitor-specific loss of Gat or Ssadh led to a significant reduction in overall cysteine levels (Fig. 2E-G, U and Fig. S2D-F). Why was cysteine level reduced in differentiated cells that do not express Gat or Ssadh RNAi?

**We thank the reviewer for this insightful observation. The reduction in cysteine levels in differentiated cells, despite RNA*i* being restricted to progenitors, suggests a non-cell-autonomous regulation of cysteine metabolism within the lymph gland. This implies that cysteine or its metabolic precursors may be synthesized predominantly in progenitor cells and subsequently shared with or influence the metabolic state of neighbouring differentiated cells.**

**This is consistent with prior findings where progenitor-derived metabolites—such as adenosine—have been shown to regulate the behaviour and fate of differentiated cells (Mondal et al. 2011). We propose that a similar inter-zonal metabolic communication exists for cysteine as well. Our data show that cysteine synthesis is highest in progenitors, and thus, perturbations in their metabolism significantly impact the overall cysteine pool across the tissue.**

**We have now clarified this interpretation in the revised text to better reflect the possibility of metabolic coupling between progenitor and differentiated cell populations. Line 197-202.**

235-245 should be in Discussion.

**This is done.**

**Reviewer #2:**

This study by Goyal and colleagues examines the metabolic mechanisms regulating glutathione biosynthesis in the larval lymph gland. Previous studies from this lab demonstrated that GABA metabolism restricts PDH activity, thus regulating flux through the TCA cycle. Here the authors demonstrate that this function of GABA metabolism influences glutathione (GSH) levels by indirectly promoting serine biosynthesis.

There are two key observations in the study. First, increased pyruvate oxidation via PDH does not restore GSH synthesis. Second, the authors present data that support a model in which pyruvate is metabolized to oxaloacetate via pyruvate carboxylase - I applaud them for using stable-isotope tracing to advance their studies. Together, these observations hint at a model in which serine biosynthesis in the lymph gland is the product of gluconeogenesis.
Overall, I find the basic premises of the work quite exciting - the authors present data on GSH metabolism in the fly lymph gland that will no doubt be used as the foundation of developmental metabolism studies for years to come - I really like this study. Below I suggest a few experiments to solidify the model prior to publication.

**We thank the reviewer for overall positive critique of our work and for finding it relevant and exciting. The suggestions made are deeply appreciated and their inclusion in the revised manuscript has made the current version very strong in terms of its completeness and the model presented. Please find our response to each comment herewith.**

1. Their model suggests that gluconeogenesis is regulating GSH production. I’d like to see knockdown of Pepck expression using RNAi, as this should similarly induce a GSH depletion.

**This is indeed a very valuable suggestion. In response, we have now performed RNA*i*-mediated knockdown of Pepck, targeting both mitochondrial (PEPCK2) and cytoplasmic (PEPCK1) isoforms, and assessed the effects on progenitor redox homeostasis.**

**Knockdown of Pepck2 (mitochondrial PEPCK) resulted in a marked reduction in GSH (Fig. 5B-C, L and Fig. S5C-D) and cysteine levels (Fig. S5M-N’ and R) specifically within the progenitor compartment. This was accompanied by increased ROS levels and a significant reduction in lymph gland size (Fig. 5D-E, M-N and Fig. S5K-L), consistent with elevated oxidative stress and in agreement with our model. These data have been added to Fig. 5 and Fig. S5 of the revised manuscript.**

**In contrast, knockdown of Pepck1 (cytoplasmic PEPCK) did not significantly affect GSH levels or lymph gland size, aside from a slight reduction compared to control (Fig. S5A-B’, P-Q). This distinction supports the specificity of mitochondrial PEPCK2 in regulating gluconeogenesis-linked GSH production in progenitor cells. This is now provided in the results section (line 369-385).**

2. Similarly, disruption of the serine biosynthetic pathway using RNA*i* in the lymph gland should result in diminished GSH production.

**We agree with the reviewer and have directly tested this by targeting the serine biosynthesis pathway using RNA*i* against Phgdh, which encodes the rate-limiting enzyme for serine production from 3-phosphoglycerate (3-PG). Knockdown of Phgdh in progenitor cells (*domeMeso>Phgdh^RNAi^)* led to a significant reduction in GSH levels (Fig. 5F, L and Fig. S5G), an increase in ROS accumulation (Fig. 5G, M and Fig. S5H), and a concomitant reduction in lymph gland size (Fig. 5N) compared to controls. These findings are consistent with our model linking serine availability to GSH synthesis and redox homeostasis in progenitors. All corresponding data are now included in Fig. 5 and Fig. S5, and in the results (line 386-393) section.**

3. Finally, the experiments described above should include a rescue, demonstrating that cysteine feeding restores GSH levels following Pepck and Phgdh RNAi.

**We appreciate the reviewer’s suggestion and have now performed rescue experiments to validate the involvement of gluconeogenesis and serine biosynthesis in GSH production. Supplementation of N-acetylcysteine (NAC), a cysteine analog, to domeMeso>Pepck2^RNAi^ and domeMeso>Phgdh^RNAi^ larvae restored progenitor GSH levels (Fig. 5H-I, L and Fig. S5I-J), reduced ROS accumulation (Fig. 5J-K, M and Fig. S5K-L), and rescued lymph gland size (Fig. 5N).**

**To further support our metabolic model, we also supplemented these RNA*i* conditions with serine. Serine supplementation successfully restored GSH (Fig. S6A-E’ and M) and cysteine (Fig. S6F-J’ and N) levels, along with lymph gland size (Fig. S6O), in both Pepck2 and Phgdh knockdown backgrounds. All corresponding data are now included in Fig. S6.**

**Additionally, given the interconversion between serine and glycine, and the role of glycine in GSH biosynthesis, we tested glycine supplementation in Phgdh*^RNAi^* larvae. Glycine feeding also led to a recovery of GSH levels relative to regular food controls (Fig. S6K-K’ and M), supporting the contribution of this pathway to redox homeostasis.**

**These data are now provided in the results section line 394-409.**

4. Are cysteine levels low because of decreased synthesis or because of elevated cystine formation, which is formed from two cysteine models in response to increased ROS. I don’t feel this experiment is absolutely necessary, but if the authors already have metabolomics data, this should be an easy comment to address.

**We thank the reviewer of bringing up this point, unfortunately we did not detect cystine with our method. This may arise as a consequence of the sensitivity of the method used for the detection of cystine. Indeed this is an intriguing point and will be followed up in our future experimentations.**

5. Please cite Flybase. This can be done in the methods using a statement such as “Flybase was used throughout the study (ref).” Such citations are key to demonstrate the importance of Flybase to US funding agencies.

**This is now added in materials and methods section (line 566-567). We apologise for the oversight on our behalf.**

In addition, I have a few minor comments:

Line 26 - GSH is undefined in the abstract. Please define the abbreviation or simply use the term glutathione in the abstract.

**This is now corrected.**

Line 64-65 - use of the word “cues” twice in the same sentence.

**This is corrected.**

Line 67 - delete the word “any” before “aberrant”. This is redundant.

**This is corrected.**

Line 133 - missing citation after “reported in literature”. Also, I think this should be reworded as “reported in the literature.”

**This is now added.**

Line 182-183 - This sentence is missing either words or commas, I’m not sure which.

**This is corrected.**

**Reviewer #3:**

In this work, the authors build upon their previous findings on GABA catabolism controlling TCA cycle activity (and the amount of ROS produced) in the Drosophila larval hematopoietic organ, the lymph gland. Here, the authors show that the suppression of TCA cycle activity via GABA catabolism allows for the production of glutathione, a tripeptide with an important role in ROS scavenging, via cysteine synthesis. The results give further insight into the mechanisms of maintaining a balance in the amount of ROS in progenitor cells. The experiments presented in the manuscript are generally well executed and back up the conclusions made. Below I present my (minor) comments on the manuscript.

**We thank the reviewer for the positive review of our work. The points raised are indeed valuable and we have addressed each one here with.**

I find the results of isotope analysis (Figure 4) to be the least convincing. For example, in Fig 4B, the OAA 13C3 amount seems to be reduced in the Gat knockdown LGs, but then looking at Fig4B’, the difference looks very minor, and even more so for citrate in Fig4C’. I’m left to wonder how much can be said based on these results/differences. Could the authors please comment on this, and edit the conclusions from this part, if necessary?

**We thank the reviewer for raising this important point and fully agree that the differences in isotopic labelling observed in Figure 4, while present, appear modest in magnitude. In light of this, we have now revised the text to reflect a more cautious interpretation of the isotope tracing results (line 335-340).**

**That said, we would like to highlight some of the technical limitations and the biological context in which these experiments were conducted. The lymph gland is a particularly small tissue, comprising roughly 50,000 cells per gland, which poses significant challenges for high-resolution metabolomics. Despite careful optimization, including reference to established literature (Allen et al. 2023; Kazek et al. 2024), the intrinsic variability and detection sensitivity contribute to the relatively high standard deviations observed. Similar levels of variability and subtle differences have been reported in metabolic profiling of small or rare cell populations.**

**Importantly, while individual metabolite differences may appear modest, we observed consistent and biologically interpretable patterns across replicates and genotypes, which informed the development of our working model.**

**To substantiate our conclusions beyond the mass spectrometry data, we conducted new genetic and functional experiments, guided by the isotopic labelling observations:**

1. **OAA Supplementation:**

**Given the proposed role of OAA as a key intermediate for gluconeogenesis, we supplemented Gat*^RNAi^* larvae with OAA. This resulted in a robust rescue of GSH (Fig. 4F-H, P and Fig. S4J-L) and cysteine (Fig. 4K-M, Q and Fig. S4O-Q) levels in progenitor cells compared to Gat*^RNAi^* alone (Fig. 4G, L and Fig. S4K, P) and moreover lymph gland size was also recovered (Fig. 4R), supporting the hypothesis that impaired OAA utilization contributes to redox imbalance.**

2. **Citrate Synthase Knockdown:**

**The increased labelling of citrate (albeit subtle) in Gat*^RNAi^* lymph glands suggested elevated TCA flux. We genetically reduced citrate synthase (Cs) activity in the Gat*^RNAi^* background, thereby limiting conversion of OAA to citrate. This led to a significant recovery of progenitor GSH (Fig. 4I, P and Fig. S4M), cysteine (Fig. 4N, Q and Fig. S4R) levels, and lymph gland size (Fig. 4R) further validating that TCA overactivity limits OAA availability for gluconeogenesis and, consequently, serine and GSH synthesis.**

**Together, these functional rescue experiments reinforce the model proposed from our isotope tracing and indicate that even modest shifts in metabolite labelling can have substantial physiological consequences in small tissues like the lymph gland.**

**We have updated the results and discussion sections to temper our claims based solely on the isotope data, while emphasizing the integrated evidence from both metabolic profiling and genetic validation.**

The statistical test used (2-way ANOVA) may not be ideal when the response/dependent variable is not continuous, but a proportion/percentage in this case. In addition, why was 2-way ANOVA used? From what I understand, it seems there is only one explanatory variable (genotype) and no interaction terms? This could be clarified.

**We thank the reviewer for raising this point and agree with the point made. We now present the data with t-test undertaken for the analysis. This has indeed made our analysis easier to understand. It was an error on our part to utilize 2-way Anova analysis considering genotype as one variable and independent batches (experimental repeats) as the other variable. We have rectified it now and applied t-test.**

Figure 1. Quantification of the ROS levels could be added.

**For Fig. 1, showing ROS data, our past work (Goyal et al. 2022), has shown that GABA catabolism through generation of succinate regulated Pdh activity and control ROS synthesis. The previous paper has detailed quantifications of the same. We have presented the concept of ROS here to bring forth the regulation on GS, also by GABA shunt, which is what we have quantified (Fig. 1L).**

**The quantifications of ROS are added in all other figures which present the new data, essentially Fig. 3N and Fig. 5M, for the data added for *domeMeso>Pepck2^RNAi^ and domeMeso>Phgdh^RNAi^* in regular food and NAC (N-acetylcysteine) supplementation conditions.**

Throughout the text, using the word “mutant” when referring to RNAi-mediated silencing is slightly misleading and could be replaced, for example, with “knockdown”.

**This is now corrected. We apologise for the erroneous use of the term “mutants”.**

Line 141 “TCA cycle activity”?

**This is corrected.**

Line 149 Sentence starting “Succinate, which is an end-product...” has a bit unclear structure and could be revised.

**This is now re-written.**

Line 182 The sentence starting “These data revealed…” is unclear and needs to be revised.

**This is revised.**

Line 222 Full name of Pdk should be given.

**Pdk is now expanded.**

Line 236 “TCA cycle”, the word “cycle” missing also elsewhere

**We have now changed TCA to TCA cycle.**

Line 292 “pyruvate” misspelled

**This is corrected.**

Line 566 What is meant by “Drosophila are not limiting”?

**We have now removed this sentence to avoid any confusions.**

**This was added as a part of statistical analysis to provide information on how sample size is decided. Since *Drosophila* are available in sufficient quantity and are not a limiting factor in an experiment, we have not performed any calculations to decide the sample size. This has been mentioned in our previous work (Goyal et al. 2022) and other literature (Hadjieconomou et al. 2020). However, in the revised draft we have deleted this sentence.**

**References:**

Allen, Annamarie E. et al. 2023. ‘Nucleotide Metabolism Is Linked to Cysteine Availability’. *Journal of Biological Chemistry* 299(4): 103039. https://linkinghub.elsevier.com/retrieve/pii/S0021925823001710.

Brazdis, Razvan-Marius et al. 2020. ‘Demonstration of Brain Region-Specific Neuronal Vulnerability in Human IPSC-Based Model of Familial Parkinson’s Disease’. *Human Molecular Genetics* 29(7): 1180–91. https://academic.oup.com/hmg/article/29/7/1180/5803130.

Chabrat, Audrey et al. 2019. ‘Pharmacological Transdifferentiation of Human Nasal Olfactory Stem Cells into Dopaminergic Neurons’. *Stem Cells International* 2019: 1–15. https://www.hindawi.com/journals/sci/2019/2945435/.

Das, Abhijit et al. 2011. ‘Identification and Analysis of a Glutamatergic Local Interneuron Lineage in the Adult Drosophila Olfactory System.’ *Neural systems & circuits* 1(1): 4. http://www.ncbi.nlm.nih.gov/pubmed/22330097.

Goyal, Manisha, Ajay Tomar, Sukanya Madhwal, and Tina Mukherjee. 2022. ‘Blood Progenitor Redox Homeostasis through Olfaction-Derived Systemic GABA in Hematopoietic Growth Control in Drosophila.’ *Development (Cambridge, England)* 149(8). http://www.ncbi.nlm.nih.gov/pubmed/34850846.

Hadjieconomou, Dafni et al. 2020. ‘Enteric Neurons Increase Maternal Food Intake during Reproduction’. *Nature* 587(7834): 455–59. https://www.nature.com/articles/s41586-020-2866-8.

Kapoor, Ankita, Achalla Padmavathi, Sukanya Madhwal, and Tina Mukherjee. 2022. ‘Dual Control of Dopamine in Drosophila Myeloid-like Progenitor Cell Proliferation and Regulation of Lymph Gland Growth’. *EMBO reports* 23(6): e52951. http://www.ncbi.nlm.nih.gov/pubmed/35476897.

Kazek, Michalina et al. 2024. ‘Glucose and Trehalose Metabolism through the Cyclic Pentose Phosphate Pathway Shapes Pathogen Resistance and Host Protection in Drosophila’ ed. Alex P. Gould. *PLOS Biology* 22(5): e3002299. https://dx.plos.org/10.1371/journal.pbio.3002299.

Kolodziejczyk, Agata, Xuejun Sun, Ian A. Meinertzhagen, and Dick R. Nässel. 2008. ‘Glutamate, GABA and Acetylcholine Signaling Components in the Lamina of the Drosophila Visual System’ ed. Patrick Callaerts. *PLoS ONE* 3(5): e2110. https://dx.plos.org/10.1371/journal.pone.0002110.

Madhwal, Sukanya et al. 2020. ‘Metabolic Control of Cellular Immune-Competency by Odors in Drosophila’ eds. Bruno Lemaitre, Anna Akhmanova, Tomas Dolezal, and Balint Z Kacsoh. *eLife* 9: e60376. https://doi.org/10.7554/eLife.60376.

Mondal, Bama Charan et al. 2011. ‘Interaction between Differentiating Cell- and Niche-Derived Signals in Hematopoietic Progenitor Maintenance’. *Cell* 147(7): 1589–1600.

Shim, Jiwon et al. 2013. ‘Olfactory Control of Blood Progenitor Maintenance’. *Cell* 155(5): 1141–53. https://linkinghub.elsevier.com/retrieve/pii/S0092867413013500.

Zhao, Yunpo et al. 2022. ‘Identification of a GABAergic Neuroblast Lineage Modulating Sweet and Bitter Taste Sensitivity’. *Current Biology* 32(24): 5354-5363.e3. https://linkinghub.elsevier.com/retrieve/pii/S0960982222016748.
